# Continuous Monitoring of Vital Signs in the General Ward Using Wearable Devices: Randomized Controlled Trial

**DOI:** 10.2196/15471

**Published:** 2020-06-10

**Authors:** Mariska Weenk, Sebastian J Bredie, Mats Koeneman, Gijs Hesselink, Harry van Goor, Tom H van de Belt

**Affiliations:** 1 Radboud University Medical Center Nijmegen Netherlands

**Keywords:** remote sensing technology, wireless technology, continuous monitoring, vital signs, wearable electronic devices, remote monitoring, digital health

## Abstract

**Background:**

Wearable devices can be used for continuous patient monitoring in the general ward, increasing patient safety. Little is known about the experiences and expectations of patients and health care professionals regarding continuous monitoring with these devices.

**Objective:**

This study aimed to identify positive and negative effects as well as barriers and facilitators for the use of two wearable devices: ViSi Mobile (VM) and HealthPatch (HP).

**Methods:**

In this randomized controlled trial, 90 patients admitted to the internal medicine and surgical wards of a university hospital in the Netherlands were randomly assigned to continuous vital sign monitoring using VM or HP and a control group. Users’ experiences and expectations were addressed using semistructured interviews. Nurses, physician assistants, and medical doctors were also interviewed. Interviews were analyzed using thematic content analysis. Psychological distress was assessed using the State Trait Anxiety Inventory and the Pain Catastrophizing Scale. The System Usability Scale was used to assess the usability of both devices.

**Results:**

A total of 60 patients, 20 nurses, 3 physician assistants, and 6 medical doctors were interviewed. We identified 47 positive and 30 negative effects and 19 facilitators and 36 barriers for the use of VM and HP. Frequently mentioned topics included earlier identification of clinical deterioration, increased feelings of safety, and VM lines and electrodes. No differences related to psychological distress and usability were found between randomization groups or devices.

**Conclusions:**

Both devices were well received by most patients and health care professionals, and the majority of them encouraged the idea of monitoring vital signs continuously in the general ward. This comprehensive overview of barriers and facilitators of using wireless devices may serve as a guide for future researchers, developers, and health care institutions that consider implementing continuous monitoring in the ward.

**Trial Registration:**

Clinicaltrials.gov NCT02933307; http://clinicaltrials.gov/ct2/show/NCT02933307.

## Introduction

### Background

Today’s technology is increasingly influencing health care [1]. Numerous wearable devices such as patches, smart watches, and even tattoos exist that can register vital signs such as heart rate (HR), respiratory rate, oxygen saturation (SpO_2_), and blood pressure (BP) [2-5]. These devices are increasingly accurate and reliable [2,6], smaller, and more user friendly than current hospital monitoring devices. This could facilitate patients’ mobility and recovery during admission [7,8]. Moreover, the devices can result in improved health outcomes and can be used as a diagnostic tool in the identification of several diseases or clinical deterioration during admission [2,9-11].

### Clinical Deterioration

Vital signs of patients in general wards are usually monitored periodically by nurses, primarily during daytime [12]. As a result, clinical deterioration in between two subsequent measurements may not always be detected and can result in unplanned admission to the intensive care unit (ICU), which is associated with longer hospital stay, increased mortality rate [13-15], and higher costs [16]. During night hours, when less medical personnel are available, clinical deterioration may remain undetected until the next morning [17]. With wearable devices, patients can be monitored more frequently or continuously. This results in additional information about a patient’s health status, particularly during out of office hours when patients are less frequently seen by nurses [4]. By implementing continuous monitoring, clinical deterioration can be detected in an earlier phase, particularly as changes in vital signs are often present 8 to 24 hours before a life-threatening event occurs [18-22]. Additional benefits of wearable device–based continuous monitoring are a reduced workload in nurses [23], improved patient comfort because of fewer vital sign measurements [8,24], and safe patient transport between wards [25]. Besides positive effects of wearable devices, continuous monitoring can lead to false alarms that result in unnecessary additional diagnostic procedures and possible alarm fatigue in health care professionals [26,27].

### Wearable Devices

Recently, ViSi Mobile (VM) and the HealthPatch (HP) were introduced to hospital care. These two wearable devices are approved by the Food and Drug Association for continuous vital sign monitoring and have shown to be as accurate as nurse measurements in admitted patients [6]. Several studies describing the opportunities of wearable devices including VM and HP were primarily focused on the accuracy of data [11]. For successful implementation in hospital wards, wearable devices for continuous monitoring of vital signs should be comfortable and user friendly for both patients and health care professionals. Besides, patients and health care professionals should be willing to use them and see the benefit of these wearable devices and of being monitored continuously. A complete overview of experiences and expectations of patients regarding continuous monitoring with wearable devices is lacking. Therefore, this study aimed to identify experiences of patients, relatives, nurses, physician assistants, and medical doctors about the use of VM and HP in daily practice for continuous monitoring of vital signs in the general ward.

## Methods

### Setting, Participants, and Sampling

This randomized controlled trial was conducted in a university hospital between April 2015 and August 2016. The objective of this study was to give an overview of the experiences and expectations regarding continuous monitoring with wearable devices by most important stakeholders. This design was chosen as a control group would give an insight into the current experiences of patients who were not yet influenced by the use of wearable devices. Besides patients, the target population consisted of nurses, physician assistants, and medical doctors who were involved in the care of the included patients. Surgical patients were included when they were scheduled for an elective abdominal surgical procedure. Patients were excluded and replaced when they were monitored for less than 24 hours. A sample size of 90 patients (45 surgical patients and 45 internal medicine patients) was estimated to be sufficient to obtain data saturation regarding interviews, based on our pilot study [6]. As there are no standards to calculate sample size for qualitative research [28], we focused on data saturation. This was defined as the moment when additional interviews would not result in new information or themes, which was discussed and decided by two experienced qualitative researchers (MW and TB). Patients’ relatives were involved if they attended the interview. We aimed to interview all nurses, physician assistants, and medical doctors who were involved in the care of the included patients to obtain a complete overview of users’ experiences and expectations. The institutional review board decided that formal approval was not required after they reviewed the study protocol extensively (Local Ethical Committee number 2015-1717). The study was conducted in accordance with The Code of Ethics of the World Medical Association (Declaration of Helsinki).

### Wearable Devices

VM (Sotera Wireless) is a patient monitoring system developed to enhance patient safety and early detection of clinical deterioration in a general ward. VM continuously measures 5-lead electrocardiography (ECG), HR, respiratory rate, SpO_2_, BP, and skin temperature. It transmits all data wirelessly to a platform with Sotera’s analytic software such as desktop PCs or tablet PCs from where health care professionals have a real-time insight into patients’ vital sign data. VM consists of a wrist device with a touch screen display that shows vital signs and a thumb sensor that measures SpO_2_ and BP. Five ECG cables and a chest sensor that measures skin temperature and respiratory rate are attached to the patient’s chest. The battery in the wrist device has to be changed every 12 to 16 hours.

The HP (Vital Connect) is a small and lightweight disposable adhesive patch that consists of two ECG electrodes and a reusable module, which contains a sensor and a Bluetooth transmitter. It contains a battery that has a wear cycle of approximately 3 to 4 days. The patch continuously measures 1-lead ECG, HR, respiratory rate, HR variability, skin temperature, steps, and body posture [29]. The patch is attached to the patient’s chest, from where it sends data via Bluetooth to a mobile device where patients can see their own vital signs. Data are transmitted to a secured Vital Connect cloud on the internet via Wi-Fi.

### Study Procedures and Data Collection

#### Interviews

Patients in the surgical and internal medicine wards provided written informed consent after being informed about the study protocol. All interviewed nurses, physician assistants, and medical doctors also signed the informed consent form. Patients were randomly assigned to (1) VM, (2) HP, or (3) control group (no device; 1:1:1). This was done to equalize individual factors between groups and minimize bias. The control group only received the regular nurse measurements. They were interviewed about their current experiences and their expectations of continuous monitoring, without being influenced by wearing a device. In the internal medicine ward, patients were randomized immediately after signing the informed consent form. Surgical patients signed the informed consent form before an elective surgical procedure and were randomized after surgery on arrival in the ward. Vital signs were continuously measured for 2 to 3 days in the VM and HP groups. Regular vital sign measurements (three times a day) by nurses continued according to the hospital protocol for all patients.

At the end of the study, patients and their relatives were interviewed face-to-face for approximately 45 min by one trained investigator. Nurses, physician assistants, and medical doctors who were involved in the care of the included patients were also interviewed. For each semistructured interview, an interview guide was used that consisted of predetermined themes based on the model for implementation by Grol and Wensing [30]. We added themes identified in a recent pilot study about monitoring with similar wearable devices [6]. Themes concerned attitude toward continuous monitoring and the wearable devices, experiences with both wearables in clinical practice, future expectations of the devices, and perception on changes in clinical care using the devices. Questions focused on, for example, feelings of safety, users’ experiences with the devices, expected effect of continuous monitoring on patient safety and quality of care, and effect on nurse-patient interaction. The interview guide is available on request. The interviews were conducted by two researchers with a biomedical and medical background and who were trained in interviewing.

#### Questionnaires

To determine psychological distress, all patients completed the short version of the State Trait Anxiety Inventory (STAI) [31,32] at baseline and on each day of the study period. On day 3, they completed the Pain Catastrophizing Scale (PCS), which provided a valid index about the extent to which people catastrophize [33]. STAI and PCS scores were compared between randomization groups as psychological distress can be a confounding factor. Furthermore, this allowed us to assess whether the devices affected psychological distress. In addition, nurses who took care of the participating patients and who were involved in, for example, attachment of the devices and changing batteries completed the System Usability Scale (SUS) [34], which is a reliable tool for assessing usability.

### Analysis

#### Interviews

All interviews were audio-recorded and transcribed verbatim. Subsequently, two researchers (MW and TB) individually performed a thematic content analysis on all data (double coding) to determine facilitators, barriers, and positive and negative effects [35,36]. The researchers discussed the results until consensus was reached. The Donabedian framework for the quality of health care was used to present all positive and negative effects [37]. This framework distinguishes structure (context in which the care is delivered), process (all actions that make up health care), and outcome (all effects on patients’ health). Facilitators and barriers were categorized according to an existing framework concerning determinants of adoption of mobile health [38,39]. New determinants regarding the use of VM and HP were added to the framework. Interviews were consecutively analyzed during the study, and saturation was assessed using histograms, in which all new factors per interview were presented. Quotes and striking issues were also documented. Once data saturation was reached, no further interviews were analyzed as it was expected that no new factors would be identified.

#### Questionnaires

STAI, PCS, and SUS scores were analyzed using SPSS package version 20.0 (SPSS Inc). STAI scores ranged from 6 to 24, and a higher score indicated more psychological distress. SUS scores ranged from 0 to 100, and a score above 68 was considered above average [34].

Descriptive statistics were presented as mean (SD). Statistical significance between patient groups regarding demographics and PCS was calculated using the analysis of variance (ANOVA) or Pearson chi-square test. ANOVA for repeated measures was used to assess differences in the STAI score between days and randomization groups. An independent samples *t* test was used to calculate the difference between HP and VM regarding SUS. STAI and SUS results were not correlated with the interview results. A *P* value less than .05 was considered significant.

## Results

### Demographics

A total of 165 patients were invited to participate: 89 patients from the surgical ward and 76 patients from the internal medicine ward. In each ward, 58 patients signed the informed consent form; 45 patients eventually participated in the study. Reasons for refusal were expectation of a large mental (n=37) or physical burden (n=10) and expected discharge within 24 hours (n=2). In the surgical ward, 13 patients were excluded because of rescheduling of the surgery (n=5), withdrawal of informed consent (n=4), early death (n=2), prolonged ICU stay (n=1), and a delirium (n=1). Reasons to exclude patients in the internal medicine ward were monitoring for less than 24 hours because of unexpected discharge (n=11) or physical burden by VM (n=2). No differences were found between randomization groups regarding age (*P*=.74) and gender (*P*=.55). Demographics are shown in Table 1. Relatives of 6 patients attended the interview. Six medical doctors (2 surgeons, 2 internists, and 2 intensivists), 3 physician assistants, and 20 nurses were interviewed.

**Table 1:** Patient demographics.

Demographics			ViSi Mobile (n=30)		HealthPatch (n=30)		Control group (n=30)	
**Gender, n (%)**								
	Male			18 (60)		22 (73)		20 (67)
	Female			12 (40)		8 (27)		10 (33)
Age (years), median (range)			63 (26-76)		56 (27-88)		62 (34-77)	
Measurement period (days) of participation in the study, median (range)			3 (1-4)		3 (1-5)		3 (2-3)	
**Reason for admission, n (%)**								
	**Colorectal disease**			8 (27)		8 (27)		5 (17)
		Malignant	7 (23)		8 (27)		5 (17)	
		Benign	1 (5)		N/A^a^		N/A	
	**Hepatobiliary disease**			5 (17)		5 (17)		5 (17)
		Malignant	5 (17)		2 (15)		5 (17)	
		Benign	N/A		3 (10)		N/A	
	**Upper gastrointestinal disease**							
		Malignant	N/A		N/A		2 (7)	
	**Neuroendocrine tumors**							
		Malignant	N/A		1 (3)		2 (7)	
	Herniation			1 (3)		1 (3)		1 (3)
	Hematological diseases			N/A		1 (3)		2 (7)
	Autoimmune diseases			4 (13)		2 (7)		N/A
	Infectious disease			3 (10)		7 (23)		6 (20)
	Other			9 (30)		5 (17)		7 (23)

^a^N/A: not applicable.

### Interview Data

**Figure 1:**
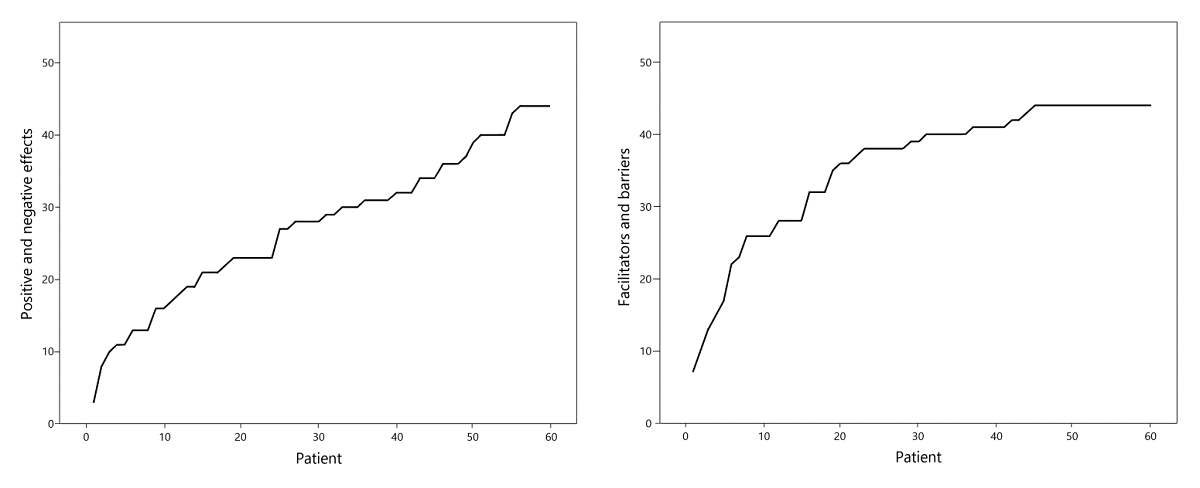
Saturation of positive and negative effects and facilitators and barriers. X-axis represents number of patients interviewed; Y-axis represents the accumulated number of new items mentioned by patients.

After analyzing 60 interviews (19 VM group, 21 HP group, and 20 control group) with patients, data saturation occurred, indicating that it was considered unlikely that new factors would be identified in additional interviews (Figure 1). We interviewed 29 health care professionals: 6 medical doctors, 3 physician assistants, and 20 nurses. After interviewing and analyzing professionals, we concluded that data saturation may not have been reached. Interviews with patients lasted for a median of 16 min (range 37 min). Generally, interviews with study participants from the control group lasted for a shorter duration as these people had no experience with the device. For professionals, interviews lasted for a median of 33 min (range 33 min). A total of 33 unique positive effects by patients and 56 positive effects by health care professionals and 14 negative effects by patients and 31 negative effects by health care professionals were identified. Patients reported 13 facilitators and 22 barriers, and health care professionals reported 13 facilitators and 36 barriers.

### Positive Effects

In the structure, process, and outcome domains, 1, 23, and 23 positive effects were identified, respectively, (Multimedia Appendix 1) by patients, their relatives, and health care professionals. Moreover, 6 patients and 2 nurses mentioned alarms as a positive effect of continuous monitoring using wearable devices. A nurse stated:

We should all receive a mini-Ipad. It can show us patients’ vital signs during our shift and will send us an alert in case the vital signs drop outside the normal ranges.

NurseID4

A total of 17 patients, 2 relatives, and 17 health care professionals expected to be able to detect clinical deterioration in an earlier phase using continuous monitoring. Moreover, 5 patients, 3 nurses, and 1 medical doctor mentioned that earlier detection can result in earlier interventions. Furthermore, 6 patients, 1 relative, and 5 nurses thought that the implementation of continuous monitoring can lead to less patient disturbances. In addition, 7 patients and 11 health care professionals thought that continuous monitoring can save time. We asked all nurses how to spend the saved time. A nurse mentioned:

Just talking to the patient. To have more time for the story of the patient.

NurseID7

Other positive effects regarding efficiency in health care were a reduced workload, shorter hospital stay, prevention of ICU admission, reduced costs, and lower amount of nursing staff required. A patient described:

You can stay shorter in the hospital and can go home with a wearable device. They can inspect your data in the hospital while you are at home. I would like that, it would feel more safe.

PatientID40

A total of 17 patients, 1 relative, and 9 health care professionals expected increased feelings of safety in patients in the general ward. In addition, patients’ relatives and nurses mentioned feeling safer. A nurse explained:

Postoperative patients have been monitored continuously at the ICU. Some do feel unsafe after return at the general ward because of a lower number of vital sign measurements.

NurseID1

All nurses and most patients encouraged the implementation of wearable devices for continuous monitoring of patients. A nurse and a patient mentioned:

This is the future. We have to deal with it and the sooner we start working with those wearable devices, the more profit we will have.

NurseID16

The future...I think only 30% of the patients will be hospitalized by then. Patients will be monitored from home with this kind of smart devices.

PatientID50

### Negative Effects

A total of 12 and 18 negative effects were identified in the process and outcome domains, respectively, by patients, their relatives, and health care professionals (Multimedia Appendix 2). Moreover, 1 patient and 5 health care professionals thought that continuous monitoring can generate an overload of information. An internist mentioned:

Sometimes you just do not want to know, making yourself crazy with too much data. Particularly when data does not influence your decision in patient’s treatment.

MedicaldoctorID5

Particularly, nurses in the surgical ward were afraid that their ward would become like an ICU; 3 nurses and 1 medical doctor thought that this can lead to reluctance in transfer to the ICU. The alarm system was mentioned as a negative effect by 3 nurses and 3 medical doctors, leading to false-positive alarms, irrelevant alarms, and alarm fatigue. Moreover, 9 patients, 1 relative, and 5 nurses were afraid that interaction between patient and health care professionals would be reduced. A patient mentioned:

You need the confidence from the nurses, I would miss that. However, quantity time might become quality time.

PatientID58

In addition, 7 nurses and 1 medical doctor mentioned that continuous monitoring would cost more time, and 1 nurse, 1 physician assistant, and 1 medical doctor thought that it would increase workload. A nurse said:

Maybe it will increase work load. What if you receive an alarm every time a patient falls asleep and the oxygen saturation decreases a little bit?

NurseID6

A total of 12 patients, 2 relatives, and 2 health care professionals mentioned that patients can become worried by being able to see their own vital signs. A patient explained:

Some people are very anxious. Like my wife...like she already said: she would overreact. I would like to know my vital signs, but she would panic.

PatientID54

### Facilitators

Eight facilitators were found in the domain Factors related to devices (Multimedia Appendix 3). In addition, 1 nurse and 1 medical doctor mentioned that by using continuous monitoring, health care professionals are able to see trends in vital signs. A surgeon stated:

Last night we saw a patient with an Early Warning Score of 3 and in the morning it suddenly was 13. Using continuous monitoring, we would have been able to see the Early Warning Score slowly increasing during the night.

MedicaldoctorID6

Moreover, 2 patients, 2 nurses, and 1 medical doctor mentioned the small size of the HP. Three patients said that they thought it was easy to view all vital signs on the VM wrist device or the mobile device of the HP. In addition, 2 patients and 1 nurse said that they think both the devices are reliable.

Three facilitators were found in the domain Individual factors. In addition, 2 patients, 2 nurses, and 2 medical doctors thought that continuous monitoring will lead to earlier detection of clinical deterioration, and 2 patients mentioned that they think that patient safety will be improved. In the Human environment domain, 8 facilitators were identified. Five patients mentioned that the devices were invisible under their clothes, and 7 patients said that they were not aware of the device. Moreover, 1 patient, 2 nurses, and 1 medical doctor mentioned fewer actions during vital sign measurements as a facilitator, such as putting on the upper arm cuff for BP measurements.

### Barriers

In the domain Factors related to the devices, 22 barriers were identified (Multimedia Appendix 3). In addition, 2 patients, 3 nurses, and 1 medical doctor mentioned the VM battery change as a barrier. VM wrist device was thought to be too big or heavy by 5 patients, 3 nurses, and 1 medical doctor. Furthermore, VM cables and the patches and electrodes were also mentioned as barriers. A patient said:

Yesterday I felt very ill. I noticed that when you do not feel very well, every line, every device is just too much.

PatientID40

Three patients mentioned that devices are not able to measure patient experiences, such as pain. A patient described:

The devices are not able to register pain. When the nurse does not visit me, I cannot tell her I am having a headache. The device will not register that.

PatientID55

Moreover, 2 patients and 5 nurses said that it is a barrier that the HP is not able to measure all vital signs. Furthermore, it was also mentioned that VM and HP both are not able to measure core temperature.

Four barriers were identified in the Individual factors domain. One medical doctor mentioned the risk of overtreatment by identifying abnormalities in vital signs that cannot be ignored. Moreover, 1 medical doctor and 1 patient said that the VM wrist device is stigmatizing. In the domain Human environment, six barriers were identified. Three patients thought that it was a burden to carry the HP mobile device. One medical doctor feared that there will be too much attention for the vital signs and less attention for the individual patient. One nurse mentioned that patients were worried that the patches would come off. Four barriers were identified in the Organizational environment domain. Two medical doctors mentioned that nurses do not have adequate training to interpret continuous data. In addition, 4 nurses and 1 medical doctor thought that there would not be enough personnel to monitor all data:

At this moment it is not feasible to monitor all patients 24 hours a day and to anticipate adequately to clinical deterioration with the amount of nursing staff we have.

NurseID6

### Questionnaires

#### Psychological Distress

No significant effect between the three randomization groups was found on STAI score (*P*=.33), and no significant within-subject effect was found in STAI score between days (*P*=.78; Table 2). Data of surgical and internal medicine patients were calculated separately; no significant effect between the randomization groups was found on STAI score (*P*=.86 and *P*=.17, respectively). No significant differences were found between the three randomization groups regarding PCS (*P*=.57; Table 2).

**Table 2:** State Trait Anxiety Inventory and Pain Catastrophizing Scale.

Group	STAI^a^ baseline, mean (SD)	STAI day 1, mean (SD)	STAI day 2, mean (SD)	STAI day 3, mean (SD)	Pain Catastrophizing Scale, mean (SD)
ViSi Mobile	11.8 (2.7)	11.3 (2.9)	10.6 (2.6)	10.6 (3.0)	14.2 (11.2)
HealthPatch	11.4 (2.7)	11.2 (2.8)	11.5 (2.8)	11.2 (3.3)	15.7 (11.6)
Control	11.0 (3.1)	11.1 (3.1)	11.2 (3.3)	11.7 (3.5)	17.4 (10.9)

^a^STAI: State Trait Anxiety Inventory.

#### Usability

The SUS was filled in by 6 nurses (3 internal medicine nurses and 3 surgical nurses), 1 for each device. Both devices scored above average, indicating good usability. No significant difference was found between VM and HP (mean 77.9, SD 18.5 and mean 82.5, SD 18.6, respectively; *P*=.68).

## Discussion

### Principal Findings

In this study, we used two wearable devices for continuous monitoring of vital signs in non-ICU patients with a wide spectrum of clinical conditions in two different wards. Our study resulted in a broad overview of experiences and expectations with the devices of both patients and health care professionals. We showed that continuous monitoring in the ward was not only well received by most patients and their relatives but also by their health care professionals. We also identified relevant barriers of continuous monitoring with wearable devices and that using wearable devices did not affect stress levels. Both patients and health care professionals expected that continuous monitoring of vital signs would lead to an earlier identification of clinical deterioration and to an improvement of quality, safety, and efficiency in health care. We also identified relevant barriers of continuous monitoring with wearable devices.

Our semistructured interviews revealed a primarily positive attitude toward continuous monitoring. A recent study by Abelson et al [40] also confirmed that surgical patients have a positive attitude toward wearable devices and mobile apps and that they are willing to use them. Earlier detection of clinical deterioration was frequently mentioned by patients and health care professionals corresponding with the findings from a recent review by Cardona-Morrell et al [10]. They showed that continuous monitoring of vital signs in the general ward leads to an earlier detection of clinical deterioration [10]. Respondents mentioned that continuous monitoring could lead to saved time and reduced workload for nurses, which is also found in other studies [10]. All nurses mentioned that they would use this time for the patient, such as mobilization, washing or showering patients, providing information, and providing a listening ear for the patient. This might solve the problem for less nurse-patient interaction, which was frequently mentioned by patients. Future research should shed light on changes in nurses’ workload after the implementation of continuous monitoring. A frequently reported barrier was the wrist device and cables of VM. Particularly, surgical patients mentioned that the VM cables were a burden in combination with other lines, such as abdominal drains and urinary catheters. However, patients did not feel restricted during daily activities. This is important as early appropriate mobilization improves recovery and reduces the risk of complications [41,42]. STAI and PCS scores revealed no differences in psychological distress between patients in the intervention and control groups, indicating that neither the VM nor the HP caused additional stress or reduced stress. According to SUS scores, the larger VM wrist device and cables did not influence the overall usability of the VM in comparison with the much smaller HP. It is expected that future devices will become even smaller while being able to wirelessly monitor an increasing number of vital signs continuously. The amount of data that will become available by continuous monitoring was mentioned as a negative effect by health care professionals, as it was expected that they would never be able to review all data. Big data analytics are available for effective storage and processing of large amounts of data [43,44]. Alarms can alert the nurse when patient’s vital signs drop out of normal ranges, resulting in a high number of false-positive or irrelevant alarms or even alarm fatigue [27,45]. Machine learning algorithms can prevent unnecessary diagnostic procedures and overtreatment because of a reduced number of irrelevant and false-positive alarms [46-48].

### Other Research

Few studies regarding continuous monitoring in the general ward exist. Brown et al [49] compared continuous monitoring using the EarlySense system with intermittent monitoring in a medical-surgical ward. This system includes a flat sensor that is placed under the patient’s bed and monitors HR and respiratory rate continuously. They found a reduced number of days in the ICU and shorter overall hospital stay because of earlier interventions in patients who were monitored continuously. However, the system is not able to monitor other vital signs such as BP, SpO_2_, and temperature when patients are out of bed. Other researchers used patches to monitor several vital signs such as the HP in this study, with promising results [50]. Using HP and VM, patients are able to mobilize throughout the hospital while being monitored continuously for relevant vital signs. VM measures almost all vital signs, which are required to calculate the Modified Early Warning Score and judge the clinical situation of the patient. More recent work found that patient monitoring systems should be tailored to users’ needs [51].

### Strengths and Limitations

An important strength of this study is that we were able to monitor patients admitted for various reasons for a longer period in a clinical setting in two different departments. We conducted a large number of semistructured interviews with both patients and health care professionals and were able to reach data saturation in patients about all predefined categories. This resulted in a comprehensive overview of the positive and negative effects of continuous monitoring and facilitators and barriers regarding VM and HP. The control group allowed us to collect current experiences from patients that were not yet influenced by using wearable devices. Regarding interviews with health care providers, data may not have saturated, although this is difficult to assess [28]. Selection bias could have occurred as not all invited patients participated in the study, particularly in the surgical ward. However, we randomized all patients to VM, HP, or a control group, and no significant differences were found between randomization groups, for example, gender, which minimized bias. Patients who refused to participate reported that they feared the mental or physical burden, particularly severely ill patients or patients with psychological distress. No differences were found in stress experienced between different randomization groups. Although the STAI questionnaire is validated for measuring psychological distress, many other stressful factors may have an impact on patients and may potentially influence the outcomes (stress before surgical procedures or complications during hospitalization).

### Future Perspectives

Implementation of wearable devices for continuous monitoring is expected to influence health care in several ways. Patient safety can be improved as trained and experienced personnel can be warned during an earlier phase of deterioration and perform early interventions. This can prevent unnecessary ICU admissions and shorten hospitality stay. Nurses will have to be trained in using wearable devices and continuous vital sign data in the general ward. It is expected that nurses will have more time for other needs of a patient during admission. Data transmission via Wi-Fi between the device and the electronic health record should be safe and accurate. Potential alarms in vital signs can be processed using predictive analytics and machine learning techniques to prevent false-positive alarming. Furthermore, patients can benefit from continuation of monitoring using the same or comparable wearable devices. Vital signs data collected at home can be shared with trained nurses or physicians. With continuous monitoring, patients can be more actively involved in their own treatment. To stimulate this, the facilitators and barriers reported in this study are of great value when planning to implement wearable devices in the general ward.

### Conclusions

According to patients and health care professionals, VM and HP have potential for continuous monitoring of vital signs in the general ward, and almost all of them encouraged the idea of monitoring vital signs continuously in the general ward. The comprehensive overview of barriers and facilitators of using wireless devices should be taken into consideration when choosing the device for implementing continuous monitoring. Continuous monitoring may facilitate the use of predictive analytics for clinical deterioration and early interventions. Further studies should explore the effect of continuous monitoring on clinical outcomes of patients in the general ward.

## Appendix

Multimedia Appendix 1 Positive effects.

Multimedia Appendix 2 Negative effects.

Multimedia Appendix 3 Facilitators and barriers.

Multimedia Appendix 4 CONSORT-eHEALTH checklist (V. 1.6.1).

## Abbreviations

ANOVA: analysis of variance
BP: blood pressure
ECG: electrocardiography
HP: HealthPatch
HR: heart rate
ICU: intensive care unit
PCS: Pain Catastrophizing Scale
SpO_2_: oxygen saturation
STAI: State Trait Anxiety Inventory
SUS: System Usability Scale
VM: ViSi Mobile
