# Supplementary material for: Continuous Monitoring of Vital Signs in the General Ward Using Wearable Devices: Randomized Controlled Trial
Source: J Med Internet Res. 2020 Jun 10;22(6):e15471. doi: 10.2196/15471 (PMC7315364; doi:10.2196/15471)
Supplement: Multimedia Appendix 1 [file jmir_v22i6e15471_app1.docx]

|  | **Patient** | **Group** | **Relatives** | | **Nurse** | **PA^a^** | | **MD^b^** | |
| --- | --- | --- | --- | --- | --- | --- | --- | --- | --- |
| **Structure** |  |  | |  |  | |  | |  |
| 1. Monitoring patients from a distance | 2 | HP^c^/Co^d^ | |  | 2 | |  | |  |
| **Process** |  |  | |  |  | |  | |  |
| 2. Vital sign monitoring | **23** |  | |  | **18** | | **4** | | **4** |
| 2.1 Monitoring patients with high MEWS |  |  | |  | 1 | |  | |  |
| 2.2 Monitoring patients who don’t call for help |  |  | |  | 1 | |  | |  |
| 2.3 More information about patients |  |  | |  |  | |  | |  |
| 2.3.1 Availability of historical data | 4 | HP/Co | |  | 2 | | 1 | | 1 |
| 2.3.2 Insight in effect of medication |  |  | |  | 1 | |  | |  |
| 2.3.3 Could assist with differential diagnosis |  |  | |  |  | |  | | 1 |
| 2.3.4 Trends | 1 | VM^e^ | |  | 5 | | 2 | | 1 |
| 2.3.5 Improved communication between physicians | 1 | Co | |  |  | |  | |  |
| 2.3.6 From home | 1 | HP | |  |  | |  | |  |
| 2.3.7 Not specified | 7 | VM/HP/Co | |  | 3 | |  | | 1 |
| 2.4 Alarms | 6 | VM/HP/Co | |  | 2 | |  | |  |
| 2.5 Data automatically in EHR |  |  | |  | 2 | | 1 | |  |
| 2.6 More reliable data |  |  | |  |  | |  | |  |
| 2.6.1 No measuring error between nurses | 1 | HP | |  |  | |  | |  |
| 2.6.2 Measurements at fixed time points | 1 | HP | |  |  | |  | |  |
| 2.6.3 Not specified | 1 | HP | |  | 1 | |  | |  |
| 3. Detection of clinical deterioration | **22** |  | | **2** | **16** | | **2** | | **5** |
| 3.1 Earlier detection of abnormal vital signs |  |  | |  |  | |  | |  |
| 3.1.1 During the night | 1 | HP | |  |  | |  | |  |
| 3.1.2 Not specified | 16 | VM/HP/Co | | 2 | 12 | | 2 | | 3 |
| 3.2 Earlier interventions |  |  | |  |  | |  | |  |
| 3.2.1 Earlier ICU admission |  |  | |  | 1 | |  | | 1 |
| 3.2.2 Not specified | 5 | VM/HP | |  | 3 | |  | | 1 |
| 4. Patient-professional interaction | **9** |  | | **1** | **12** | |  | | **1** |
| 4.1 Less patient disturbances | 6 | VM/HP/Co | | 1 | 5 | |  | |  |
| 4.2 More contact between nurse and patient | 2 | VM | |  | 2 | |  | |  |
| 4.3 Less actions during MEWS measurements |  |  | |  |  | |  | |  |
| 4.3.1 More hygiene by not touching the patient |  |  | |  | 1 | |  | |  |
| 4.3.2 Not specified | 1 | VM | |  | 4 | |  | | 1 |
| 5. Increased patient mobility |  |  | |  | 2 | | 1 | | 2 |
| **Outcome** |  |  | |  |  | |  | |  |
| 6. Quality and safety | **8** |  | |  | **10** | |  | |  |
| 6.1 Improvement of quality of care | 1 | Co | |  | 3 | |  | |  |
| 6.2 Improvement of patient safety | 7 | VM/HP/Co | |  | 7 | |  | |  |
| 7. Efficiency in health care | **14** |  | |  | **17** | | **3** | | **7** |
| 7.1 Time saving |  |  | |  |  | |  | |  |
| 7.1.1 Time for other activities | 1 | HP | |  |  | | 1 | | 1 |
| 7.1.2 Particularly during evening and night shifts |  |  | |  | 1 | |  | | 1 |
| 7.1.3 Not specified | 6 | VM/HP/Co | |  | 7 | |  | |  |
| 7.2 Reduced work load | 3 | HP | |  | 5 | | 1 | | 1 |
| 7.3 Shorter hospital length of stay |  |  | |  |  | |  | |  |
| 7.3.1 Earlier discharge with HP | 1 | HP | |  |  | |  | |  |
| 7.3.2 Shorter ICU length of stay |  |  | |  |  | |  | | 1 |
| 7.3.3 Not specified |  |  | |  |  | | 1 | | 1 |
| 7.4 Prevention of ICU admission |  |  | |  | 4 | |  | |  |
| 7.5 Reduced costs | 2 | HP/Co | |  |  | |  | | 1 |
| 7.6 Less nursing staff needed | 1 | HP | |  |  | |  | |  |
| 7.7 Not specified |  |  | |  |  | |  | | 1 |
| 8. Psychosocial domains/well being | **24** |  | | **4** | **9** | | **1** | | **3** |
| 8.1 Feelings of safety patient | 17 | VM/HP/Co | | 1 | 5 | | 1 | | 3 |
| 8.2 Feelings of safety nurse | 1 | Co | |  | 1 | |  | |  |
| 8.3 Feelings of safety relatives | 1 | Co | | 3 |  | |  | |  |
| 8.4 More privacy |  |  | |  | 1 | |  | |  |
| 8.5 More rest in the room | 2 | HP/Co | |  |  | |  | |  |
| 8.6 Better sleep at night | 3 | HP/Co | |  | 2 | |  | |  |
| 9. Insight in own vital sign monitoring | **4** |  | | **1** | **2** | |  | |  |
| 9.1 Patients are more involved in own treatment | 1 | Co | |  | 1 | |  | |  |
| 9.2 To be relieved | 2 | VM | | 1 |  | |  | |  |
| 9.3 Specified | 1 | Co | |  | 1 | |  | |  |
| 10. No restriction in daily activities | 1 | HP | |  |  | |  | |  |

Multimedia Appendix 1. Positive effects.

^a^PA, Physician assistant; ^b^MD, Medical doctor; ^c^HP, HealthPatch; ^d^Co, Control group; ^e^VM, ViSi Mobile.
