# Supplementary material for: Continuous Monitoring of Vital Signs in the General Ward Using Wearable Devices: Randomized Controlled Trial
Source: J Med Internet Res. 2020 Jun 10;22(6):e15471. doi: 10.2196/15471 (PMC7315364; doi:10.2196/15471)
Supplement: Multimedia Appendix 2 [file jmir_v22i6e15471_app2.docx]

|  | **Patient** | **Group** | **Relatives** | **Nurse** | **PA^b^** | **MD^c^** |
| --- | --- | --- | --- | --- | --- | --- |
| **Process^a^** |  |  |  |  |  |  |
| 1. Vital sign monitoring | **1** |  |  | **12** | **1** | **5** |
| 1.1 Overkill information |  |  |  |  |  |  |
| 1.1.1 Things that cannot be ignored |  |  |  | 2 |  |  |
| 1.1.2 Less attention for vital signs |  |  |  | 1 |  |  |
| 1.1.3 Not specified | 1 | HP^d^ |  | 1 |  | 1 |
| 1.2 More similarities with ICU |  |  |  |  |  |  |
| 1.2.1 Delayed admission to ICU |  |  |  | 3 |  | 1 |
| 1.2.2 Not specified |  |  |  | 2 | 1 |  |
| 1.3 Alarms |  |  |  |  |  |  |
| 1.3.1 False positive alarms, e.g. movements |  |  |  | 1 |  | 1 |
| 1.3.2 Irrelevant alarms, particularly during night |  |  |  | 2 |  | 1 |
| 1.3.3 Alarm-fatigue |  |  |  |  |  | 1 |
| 2. Interaction between professionals and patients | **9** |  | **1** | **5** | **1** |  |
| 2.1 Less nurse-patient contact |  |  |  |  |  |  |
| 2.1.1 Less use of clinical eye |  |  |  | 2 |  |  |
| 2.1.2 Not specified | 9 | VM^e^/HP/Co^f^ | 1 | 3 |  |  |
| 2.2 More nurse-physician contact |  |  |  |  | 1 |  |
| 3. Reduced patient mobility | 1 | Co |  |  | 1 |  |
| **Outcome** |  |  |  |  |  |  |
| 4. Efficiency in health care | **1** |  |  | **9** | **1** | **2** |
| 4.1 Costs more time |  |  |  |  |  |  |
| 4.1.1 More time with computer |  |  |  | 4 |  | 1 |
| 4.1.2 To connect patients with devices |  |  |  | 2 |  |  |
| 4.1.3 Interns need more explanations |  |  |  | 1 |  |  |
| 4.2 Increased workload |  |  |  |  |  |  |
| 4.2.1 More questions from patients |  |  |  | 1 | 1 |  |
| 4.2.2 Not specified |  |  |  |  |  | 1 |
| 4.3 Unnecessary treatments | 1 | Co |  | 1 |  |  |
| 5. Psychosocial domains/well-being | **16** |  | **3** | **9** | **1** | **1** |
| 5.1 Obsessed patient | 1 | VM | 1 | 5 |  |  |
| 5.2 Worried patient |  |  |  |  |  |  |
| 5.2.1 Wrong interpretation of vital signs | 1 | HP | 1 |  | 1 |  |
| 5.2.2 Certain patient groups (e.g. anxiety) | 1 | VM |  |  |  |  |
| 5.2.3 By hearing alarms | 1 | HP |  |  |  |  |
| 5.2.4 That nobody is watching vital signs | 1 | Co |  |  |  |  |
| 5.2.5 Not specified | 8 | VM/Co | 1 | 1 |  |  |
| 5.3 Increased feelings of illness |  |  |  | 1 |  |  |
| 5.4 False sense of safety | 1 | HP |  | 1 |  | 1 |
| 5.5 Feelings of unsafety | 1 | Co |  |  |  |  |
| 5.6 Worried family |  |  |  | 1 |  |  |
| 6. Restriction in daily activities | 1 | Co |  |  |  |  |
| 7. Reduced patient empowerment | 1 | Co |  |  |  |  |

Multimedia Appendix 2. Negative effects.

^a^No negative effects in the “Structure” field were found; ^b^PA, Physician assistant; ^c^MD, Medical doctor; ^d^HP, HealthPatch; ^e^VM, ViSi Mobile; ^f^Co: Control group.
