# Supplementary material for: Continuous Monitoring of Vital Signs in the General Ward Using Wearable Devices: Randomized Controlled Trial
Source: J Med Internet Res. 2020 Jun 10;22(6):e15471. doi: 10.2196/15471 (PMC7315364; doi:10.2196/15471)
Supplement: Multimedia Appendix 3 [file jmir_v22i6e15471_app3.docx]

|  | **Fac** | **Bar** | **Device** | **Patient** | **Nurse** | **PA** | **MD** |
| --- | --- | --- | --- | --- | --- | --- | --- |
| **1. Factors related to devices** |  |  |  |  |  |  |  |
| 1.1 Design and technical concerns |  |  |  |  |  |  |  |
| 1.1.1 WiFi connection between VM and Toughbook^a^ |  | 2 | VM^d^ |  | 1 | 1 |  |
| 1.1.2 Artifacts in data^a^ |  | 3 | VM/HP^e^ | 1 | 1 |  | 1 |
| 1.1.3 Data too much/unclear^a^ |  | 2 | VM/HP |  |  |  | 2 |
| 1.1.4 Able to see trends^a^ | 2 |  | VM/HP |  | 1 |  | 1 |
| 1.1.5 Able to see vital signs from a distance^a^ | 1 |  | VM/HP |  | 1 |  |  |
| 1.1.6 Small size^a^ | 5 |  | HP | 2 | 2 |  | 1 |
| 1.1.7 Battery change^a^ |  | 5 | VM | 2 | 3 |  |  |
| 1.1.8 Wrist device^a^ |  |  |  |  |  |  |  |
| 1.1.8.1 Too big/heavy |  | 9 | VM | 5 | 3 |  | 1 |
| 1.1.8.2 Not easy to read vital signs |  | 1 | VM |  | 1 |  |  |
| 1.1.8.3 Light turns on during the night |  | 3 | VM | 3 |  |  |  |
| 1.1.8.4 Too loose |  | 1 | VM | 1 |  |  |  |
| 1.1.8.5 Too tight |  | 1 | VM | 1 |  |  |  |
| 1.1.9 Cables^a^ |  | 14 | VM | 8 | 6 |  |  |
| 1.1.10 Patches/electrodes^a^ |  | 16 | VM/HP | 9 | 6 |  | 1 |
| 1.1.11 No upper arm cuff^a^ | 1 |  | VM/HP |  | 1 |  |  |
| 1.1.12 Restriction during daily activities^a^ |  | 7 | VM | 2 | 5 |  |  |
| 1.1.13 Alarms (irrelevant/false-positive)^a^ |  | 12 | VM/HP | 2 | 7 | 1 | 2 |
| 1.2 Characteristics of the innovation |  |  |  |  |  |  |  |
| 1.2.1 Perceived usefullness |  |  |  |  |  |  |  |
| 1.2.1.1 Devices have no clinical eye^a^ |  | 3 | VM/HP | 1 |  |  | 2 |
| 1.2.1.2 Not able to measure with devices during diagnostic procedures^a^ |  | 3 | VM/HP | 1 | 2 |  |  |
| 1.2.1.3 Devices are not able to measure patient experience (e.g. pain)^a^ |  | 3 | VM/HP | 3 |  |  |  |
| 1.2.2 Perceived ease of use |  |  |  |  |  |  |  |
| 1.2.2.1 Connecting patients^a^ | 1 | 2 | VM |  | 3 |  |  |
| 1.2.2.2 Display/interface (VM wrist device/HP mobile device)^a^ | 3 |  | VM/HP | 3 |  |  |  |
| 1.3 System reliability | 3 |  | VM/HP | 2 | 1 |  |  |
| 1.4 Legal issues |  |  |  |  |  |  |  |
| 1.4.1 Confidentiality – privacy concerns |  | 2 | VM/HP | 2 |  |  |  |
| 1.5 Validity of the resources |  |  |  |  |  |  |  |
| 1.5.1 Satisfaction about content available (completeness) |  |  |  |  |  |  |  |
| 1.5.1.1 Not able to measure all vital signs^a^ |  | 7 | HP | 2 | 5 |  |  |
| 1.5.1.2 Measures skin temperature instead of core temperature^a^ |  | 1 | VM | 1 |  |  |  |
| 1.5.2 Accuracy | 4 | 2 | VM/HP | 4 | 2 |  |  |
| 1.6 Cost issues |  | 1 | VM/HP |  |  |  | 1 |
| **2. Individual factors: knowledge, attitude, socio-demographic characteristics** |  |  |  |  |  |  |  |
| 2.1 Attitude |  |  |  |  |  |  |  |
| 2.1.1 Agreement with the devices |  |  |  |  |  |  |  |
| 2.1.1.1 Time consuming/time saving |  |  |  |  |  |  |  |
| 2.1.1.1.1 Battery change^a^ |  | 2 | VM |  | 2 |  |  |
| 2.1.1.1.2 Too much data (reviewing all the data take too much time)^a^ |  | 2 | VM/HP |  | 1 | 1 |  |
| 2.1.1.2 Outcome expectancy (use leads to desired outcome) |  |  |  |  |  |  |  |
| 2.1.1.2.1 Earlier detection of abnormal vital signs^a^ | 6 |  | VM/HP | 2 | 2 |  | 2 |
| 2.1.1.2.2 Earlier discharge with HP^a^ | 2 |  | VM/HP | 1 |  | 1 |  |
| 2.1.1.2.3 Improvement of patient safety^a^ | 2 |  | VM/HP | 2 |  |  |  |
| 2.1.1.2.4 Overtreatment of patients^a^ |  | 1 | VM/HP |  |  |  | 1 |
| 2.1.1.3 Motivation/resistance to use |  |  |  |  |  |  |  |
| 2.1.1.3.1 Wrist device is stigmatizing^a^ |  | 2 | VM | 1 |  |  | 1 |
| **3. Human environment** |  |  |  |  |  |  |  |
| 3.1 Factors associated with patients |  |  |  |  |  |  |  |
| 3.1.1 Patients’ attitudes and preferences regarding devices |  |  |  |  |  |  |  |
| 3.1.1.1 Able to see own vital signs^a^ | 3 |  | VM/HP | 3 |  |  |  |
| 3.1.1.2 Device invisible under clothes^a^ | 5 |  | VM/HP | 5 |  |  |  |
| 3.1.1.3 Not aware of device^a^ | 7 |  | VM/HP | 7 |  |  |  |
| 3.1.1.4 Extra device with HP^a^ |  | 3 | HP | 3 |  |  |  |
| 3.1.1.5 Wanted to stop wearing VM, my wrist are not that well^a^ |  | 1 | VM | 1 |  |  |  |
| 3.1.1.6 Short battery life^a^ |  | 1 | VM | 1 |  |  |  |
| 3.1.1.7 Increased patient comfort (vitals measured with one device)^a^ | 1 |  | VM | 1 |  |  |  |
| 3.1.2 Patient/health professional interaction |  |  |  |  |  |  |  |
| 3.1.2.1 Less attention for patient^a^ |  | 1 | VM/HP |  |  |  | 1 |
| 3.1.2.2 Less nurse-patient contact^a^ |  | 1 | VM/HP | 1 |  |  |  |
| 3.1.2.3 Less patient disturbances during the night^a^ | 3 |  | VM/HP | 2 |  |  | 1 |
| 3.1.2.4 Less actions during vital sign measurements^a^ | 4 |  | VM/HP | 1 | 2 |  | 1 |
| 3.1.3 Other factors associated with patients |  |  |  |  |  |  |  |
| 3.1.3.1 Patients were worried that patches would come off^a^ |  | 1 | VM |  | 1 |  |  |
| 3.2 Factors associated with healthcare providers^a^ |  |  |  |  |  |  |  |
| 3.2.1 Device localized at chest^a^ | 1 |  | HP |  | 1 |  |  |
| 3.2.2 Feelings of safety^a^ | 1 |  | VM/HP |  | 1 |  |  |
| **4. Organisational environment** |  |  |  |  |  |  |  |
| 4.1 Internal environment |  |  |  |  |  |  |  |
| 4.1.1 Work (nature of work) |  |  |  |  |  |  |  |
| 4.1.1.1 Time constraints and workload |  | 1 | VM/HP |  | 1 |  |  |
| 4.1.2 Skill – Staff |  |  |  |  |  |  |  |
| 4.1.2.1 Staff issues (stability, shortage) |  |  |  |  |  |  |  |
| 4.1.2.1.1 Not enough personel to monitor all data^a^ |  | 5 | VM/HP |  | 4 |  | 1 |
| 4.1.3 Organisational factors |  |  |  |  |  |  |  |
| 4.1.3.1 Training/lack of or inadequate training |  |  |  |  |  |  |  |
| 4.1.3.1.1 Nurses are not able to anticipate to deteriorating vital signs^a^ |  | 1 | VM/HP |  |  |  | 1 |
| 4.1.3.1.2 Nurses cannot handle fluctuations in vital signs^a^ |  | 1 | VM/HP |  |  |  | 1 |

Multimedia Appendix 3. Facilitators and barriers

^a^These items are added to the Gagnon framework. ^b^PA, Physician assistant; ^c^MD, Medical doctor; ^d^VM, ViSi Mobile; ^e^HP, HealthPatch.
